# The Construction of Suersen’s Obturators

**Published:** 1885-04

**Authors:** Th. Weber

**Affiliations:** Helsingfors, Finland


### THE CONSTRUCTION OF SUERSEN'S OBTURATORS.

BY DR. TH. WEBER, HELSINGFORS, FINLAND,

It is not the intention of the writer to say much about his own experience in this field of dentistry, but to confine himself chiefly to the work of his esteemed teacher, the Geheimer Hofrath Dr. Wilhelm Suersen, of Berlin, who has done a great deal for the advancement of oral prosthesis, as well as dental science, in Germany. It was a source of regret to find that in this country Suersen's valuable inventions were (with few exceptions) comparatively unknown; I therefore regard it as my duty to him, as well as to the dental profession of this country, to publish his method of constructing obturators. Dr. Suersen is not alone a man of great scientific attainments, but he is also most kind and liberal in the practical application of his knowledge; he is ever willing to impart advice and give information to dental practitioners, whether to him known or unknown. He especially shows his charitable disposition when poor sufferers apply to him for aid in treatment, often supplying them with appliances without remuneration of any kind. It is not, therefore, to be wondered at, that he occupies one of the most prominent positions in the dental profession of Germany. At the meeting of the Central Verein Deutscher Zahnarzte, held at Hamburg, August the 6th, 7th and 8th, 1867 (Deutsch. Vierjahrsschr. f. Zahnheilkunde, 1867, page 269, etc.), Dr. Suersen demonstrated his ingenious method of constructing obturators entirely of hard rubber, to the satisfaction of all the members present.

Palatine defects are either congenital or acquired, and each case of either class requires different treatment. While the acquired lesions only need a perfect fitting apparatus to enable the patient to articulate perfectly in a very short space of time, a patient with a congenital defect will need constant instruction in articulation for weeks or months, even with the most perfect appliance that can be made. A patient with a congenital defect, for whom an obturator has just been inserted, is like an infant; he is unable to control his muscles in such a manner as to produce perfect articulate sounds, especially when he is advanced in life. In these cases the patient tries to imitate the sounds of other persons as nearly as possible, thus finally using the muscles of the palate and the tongue in an abnormal way.

Staphylorrhaphic and Urnoplastic operations have never given good results; at least an improvement in articulation has seldom or never been obtained, and they are, therefore, discarded by most modern good surgeons. When a velum has been united by an operation, it generally becomes too short to enable it to come in contact with the superior constrictor muscles of the pharynx, and the nasal passages in relation to speech are in nearly the same condition that they were before the operation.

Although I take it for granted that every one who attempts to restore palatine defects is acquainted with the anatomy and physiology of the parts, I shall briefly refer to some peculiarities of the more important muscles connected with articulation. During articulation the posterior nares are more or less closed by the soft palate, except in the case of the two letters M and N, when the air must pass directly from the larynx through the nose. In the pronunciation of all the other consonants, however, the posterior nares must be closed in such a manner that all the air is obliged to pass through the mouth. The vowels are pronounced in a similar manner, although in distinct articulation some air does pass through the nasal passages, but without producing a nasal sound. The amount of air passing through the nose varies with each of the vowels. In sounding the letter A, for instance, more air passes through the nose than with O or U, and still less with E and I. If, therefore, the nasal passages are stopped up by an inflammation of the Schneiderian membrane, a polypus, or an obturator which extends too high into the nasal cavity, the letters M and N can only be pronounced with great difficulty, and the vowels will be accompanied by a nasal sound.

Articulation, therefore, is produced by a continuous opening and closing of the nasal passages, through the muscles of the soft palate, together with the superior constrictor of the pharynx. The latter muscle contracts with the pronunciation of every letter, to which fact Dr. Suersen was the first to draw attention, and it is this muscle in particular upon which the whole success of a Suersen's obturator depends.

The elastic artificial vela, invented by Dr. Stern, and afterwards modified by Dr. N. W. Kingsley in such a very ingenious manner, give most beautiful results, but they cannot be employed in every defect. In acquired cases, where the cleft is very wide and the levator and tensor palati muscles are destroyed, an elastic velum is seldom applicable. Another serious objection to the use of soft rubber in the mouth is its rapid deterioration, thus giving rise to putrefactive changes, which often cause inflammatory reactions in the parts with which they are in contact.

(TO BE CONTINUED.)

